# Smokeless tobacco (*paan* and *gutkha*) consumption, prevalence, and contribution to oral cancer

**DOI:** 10.4178/epih.e2017009

**Published:** 2017-03-09

**Authors:** Kamal Niaz, Faheem Maqbool, Fazlullah Khan, Haji Bahadar, Fatima Ismail Hassan, Mohammad Abdollahi

**Affiliations:** 1 International Campus, Tehran University of Medical Sciences (IC-TUMS), Tehran, Iran; 2 Department of Toxicology and Pharmacology, Faculty of Pharmacy, Tehran University of Medical Sciences, Tehran, Iran; 3 Toxicology and Diseases Group, Pharmaceutical Sciences Research Center, Tehran University of Medical Sciences, Tehran, Iran; 4 Institute of Paramedical Sciences, Khyber Medical University, Peshawar, Pakistan

**Keywords:** Fibrosis, Oral cancer, Areca, Smokeless tobacco, Prevalence

## Abstract

Smokeless tobacco consumption, which is widespread throughout the world, leads to oral submucous fibrosis (OSMF), which is a long-lasting and devastating condition of the oral cavity with the potential for malignancy. In this review, we mainly focus on the consumption of smokeless tobacco, such as *paan* and *gutkha*, and the role of these substances in the induction of OSMF and ultimately oral cancer. The list of articles to be examined was established using citation discovery tools provided by PubMed, Scopus, and Google Scholar. The continuous chewing of *paan* and swallowing of *gutkha* trigger progressive fibrosis in submucosal tissue. Generally, OSMF occurs due to multiple risk factors, especially smokeless tobacco and its components, such as betel quid, areca nuts, and slaked lime, which are used in *paan* and *gutkha*. The incidence of oral cancer is higher in women than in men in South Asian countries. Human oral epithelium cells experience carcinogenic and genotoxic effects from the slaked lime present in the betel quid, with or without areca nut. Products such as 3-(methylnitrosamino)-proprionitrile, nitrosamines, and nicotine initiate the production of reactive oxygen species in smokeless tobacco, eventually leading to fibroblast, DNA, and RNA damage with carcinogenic effects in the mouth of tobacco consumers. The metabolic activation of nitrosamine in tobacco by cytochrome P450 enzymes may lead to the formation of N-nitrosonornicotine, a major carcinogen, and micronuclei, which are an indicator of genotoxicity. These effects lead to further DNA damage and, eventually, oral cancer.

## INTRODUCTION

The term “smokeless tobacco” refers to the consumption of unburned tobacco, in the form of chewing, spitting, dipping, and snuff. Consumers chew the tobacco in the mouth and spit out the juice that builds up. Nicotine and other constituents are absorbed in the lining of oral cavity. People of many regions, including India, Pakistan, other Asian countries, and North America, have a long history of smokeless tobacco use. Approximately 28 chemical constituents present in smokeless tobacco are carcinogenic in nature, among which nitrosamine is the most prominent [1].

People mostly use *paan* and *gutkha* due to a lack of awareness and education. They are not aware of the harmful effects associated with the use of these substances, and it has been reported that these products are consumed for perceived beneficial effects, such as mouth freshening, aid in digestion, germ-killing, astringency, mood enhancement, tension relief, and oral cleaning [1]. *Gutkha* is sweet in taste, and children consider it to be a form of candy. Many people believe that *gutkha* is a mouth freshener, but its pleasant taste and sweetness aggregate microbes, causing damage to teeth. The use of *paan* and *gutkha* is difficult to control in most countries where it is widespread, and their extensive use leads to oral cancer [2]. The consumption of smokeless tobacco and areca nut is high in South Asian countries in the form of *paan*. In various South Asian languages, *paan* simply means “leaf.” Various ingredients are wrapped in the betel leaf. The common components of *paan* are tobacco, seeds, quenched lime, spices, and areca nut enfolded in betel quid [2]. In many developed and developing countries, tobacco is widely used with other constituents, as shown in Table 1. Over three decades ago, a tobacco industry emerged in India producing *gutkha*, which consists of slaked lime, areca nut, chewing tobacco, spices, and catechu packed in tins or pouches [2].

Oral submucous fibrosis (OSMF) is a persistent disorder of the oral cavity characterized by irritation and progressive fibrosis of the superficial and deep connective tissues. Oral cancer has been commonly observed in India, Pakistan, Sri Lanka, Taiwan, China, Indonesia, and Malaysia [3,4]. It is believed that the pathogenesis of OSMF is multifactorial, and is associated with nutritional deficiencies; the consumption of smokeless areca nuts, chilies, and lime; genetic abnormalities; betel quid; tobacco smoking; herpes simplex virus; human papilloma virus (HPV); chronic candidiasis; and immunological depression [5]. Oral cancer is the sixth most predominant type of cancer worldwide, affecting both genders equally, although it is particularly common in men in developing countries [6].

Smokeless tobacco and areca nut consumption in various forms is part of traditional culture in the US and in South Asian countries. Essentially, areca nut is the fruit derived from *Areca catechu*. It is produced in a chewable form and is also the main component of various products used daily by the younger population. Areca nut contains tannins (11 to 26%), which act as stimulants, and alkaloids (0.15 to 0.67%), mainly arecoline [2].

Areca nuts are extensively used, and have noxious and stimulant effects. Studies have reported that areca nut leads to diminished hunger, enhanced digestion, altered concentration, and relaxation, and sometimes also increases alertness [2]. The use of *gutkha* has been shown to have genotoxic and clastogenic properties [1]. In some cases, alcohol plays an important role in oral cancer, rather than smoking [7]. The use of smokeless tobacco products together with alcoholism and smoking increases the chance of oral cancer. In this study, our main focus was on the consumption of *paan* and *gutkha*, which are common smokeless products, and their role in the induction of OSMF, which ultimately leads to oral cancer.

## METHODS

A bibliographic search for the current review was conducted on PubMed, Scopus, and Google Scholar for articles on oral cancer due to *paan* and *gutkha* consumption. First, a PubMed search was conducted for the following terms: “presence of smokeless tobacco in Taiwan,” “prevalence in India,” “prevalence in Pakistan,” “presence of smokeless in Asia,” “occurrence of *paan* and *gutkha* in Tanzania,” “epidemiological prevalence and presence of smokeless tobacco in Indonesia and Malaysia,” “prevalence and presence of *paan* and *gutkha* in Cambodia,” and “prevalence of *paan* and *gutkha* in migrated peoples.”

**Figure 1.**
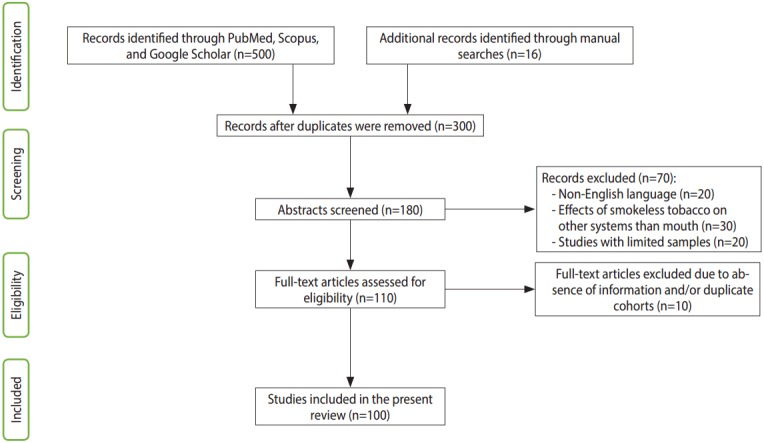
Flow diagram of the included studies. The flow chart presents the number of citations and resources that were screened, excluded, and/or included in the review.

Scopus was searched for terms including: “oral cancer due to *paan* and *gutkha*,” “genotoxic effect of *paan*,” “*paan* and *gutkha* chewers,” “betel quid compounds,” “risk factor of oral cancer,” “prevalence of oral cancer,” “*paan* associated with oral cancer,” “smokeless products lead to oral cancer,” and “*gutkha* usage.” Google Scholar was searched for keywords such as “carcinogenic effect of betel,” “mechanism of oral cancer due to *paan* and *gutkha*,” “ban of *paan* and *gutkha*,” and “OSMF mechanism and etiopathogenesis.” The data included in this article were filtered to ensure that they were specifically related to human beings and some laboratory animals. Most studies that investigated the toxicity of tobacco in other systems were excluded. Furthermore, articles were excluded if the data on oral cancer were associated with other brain and neck cancers. Studies that focused on single or limited cases with adverse effects but did not show a clear role of smokeless tobacco in the pathogenesis were also excluded. Eventually, a total of 90 reports indexed in Google Scholar and/or PubMed were found to satisfy the inclusion criteria. Studies not indexed in PubMed were obtained by manual searching in Google Scholar, and 10 such reports that satisfied the inclusion criteria were additionally retrieved. Therefore, a total of 100 studies were included in this review (Figure 1).

## GEOGRAPHIC PREVALENCE OF USAGE OF SMOKELESS TOBACCO PRODUCTS AND ORAL CANCER

The habit of chewing areca nut in various products has been reported in many countries, such as Thailand, Sri Lanka, Bangladesh, Pakistan, Malaysia, Cambodia, China, Indonesia and New Guiana. In addition, it is also widespread in the migrant populations in places such as the UK, South and East Africa, Australia, and North America [4]. In New Guiana, people use betel quid separately, with lime kept in the commissure of the mouth [8]. In Southeast Asian countries, tobacco is often used with betel quid, and smoking is also common. Inhabitants of the mountains of Cambodia, Myanmar, Thailand, and Laos add areca to the roots of other local plants, such as cinnamon and cloves, in the betel quid for consumption [9]. Approximately 390,000 cases of oral and/or pharyngeal cancers occur annually worldwide, of which around 58% are in South and Southeast Asian countries [2]. Some countries in which smokeless tobacco is consumed are discussed below.

### Taiwan

Traditionally, tobacco has been consumed as part of the culture in some countries of the world. However, in Taiwan, an increase in the consumption of *paan*, *gutkha* and other smokeless tobacco products has been reported, especially among children and teenagers. Many epidemiological studies have been conducted in Taiwan, where betel leaves or betel inflorescence is used with areca nut. The prevalence of smokeless tobacco use among men and women is 9.8 and 1.6%, respectively [10]. In 1991, a survey-based study was conducted among the residents of Kaohsiung in Taiwan; among 1,162 individuals aged 15 years and above, 13.3% consumed betel quid and 2.8% were daily chewers [11]. The increase in the usage of betel quid has been investigated in many studies. The phenomenon appears due to the surplus in markets and shops selling ready-made quid. In more than 53% of cases, use of these products started among family members influenced by the grandfather and father [10]. In another school-based survey in Taiwan, the consumption of betel quid was higher, especially among boys rather than girls. It was also common amongst those who used to drink alcohol or smoke tobacco [10,12]. Betel quid use was common in professional schools rather than senior or junior high-school [12,13].

### India

According to the National Report of Global Adult Tobacco Survey conducted in India and Bangladesh, the current prevalence of smokeless tobacco use is 25.9 and 27.2%, respectively. There are 30 different types of smokeless products available in these countries, including *zarda*, which contains dried and boiled tobacco leaves, limes, areca nut, additives, spices, and tannins [14]. Oral cancer accounts for 30 to 40% of cancer cases reported in India, and the most obvious cause is the extensive use of tobacco products, consumed via smoking and/or smokeless chewing products [15]. In addition, oral cancer occurrence is especially high in Uttar Pradesh in north India due to the extraordinary rate of consumption of smokeless tobacco products, such as *paan* and *gutkha* [16].

In India, the prevalence of oral cancer is high. It has been previously documented that besides other factors, the extensive use of *paan*, *gutkha*, and *zarda* could also contribute to the development of oral cancer [2]. In India, mostly children and teenagers chew *gutkha* occasionally or regularly. In Mumbai, 40% of school students and 70% of college students have been reported to regularly consume *gutkha*. Although some states of India have banned *gutkha* consumption due to its carcinogenic properties and other hazardous effects, it is still actively sold on the black market [2]. In addition, the widespread habit of *paan* and *gutkha* use is not limited to the Indian subcontinent, but extends to immigrants living in US and Europe [17-20]. In the Indian city of Wardha, *gutkha* was found to be used by approximately 46.4% of men and 20% of women [18].

### Pakistan

After India, Pakistan is the second prominent country in which these smokeless tobacco products are consumed, with a prevalence among Pakistani men and women of 21.3 and 19.3%, respectively. More than 90% of oral cancer cases have been reported to be associated with the use of tobacco products, indicating that they are vital factors triggering oral cancer. A study reported that women who chewed tobacco more than 10 times a day had a higher risk of oral cancer than non-tobacco chewers [21]. A study conducted by Muwonge et al. [22] reported that smokeless tobacco users were at a higher risk of oral cancer, along with other abnormalities, than tobacco smokers.

Worldwide, almost 600 million people consume areca nut as part of traditions and/or everyday life. Many epidemiological studies in India and Pakistan have reported that 3.3 to 37.0% of people chewed *paan*. In Pakistan and India, oral cancer is the most common type of cancer, after breast and lung cancer. Breast cancer is mostly observed among women [23]. The incidence of mouth, tongue, hypopharynx, nasopharynx, and lip cancer was found to be equal between men and women in Karachi, Pakistan [16,24-26].

The use of these products is considered to be a normal cultural practice. *paan*, *gutkha*, *chaalia*, *naswar*, and *toombak* are widely used, but ultimately lead to OSMF and oral cancer. Various studies have suggested that in Pakistan, India, and Nepal, 20 to 30% of adults and teenagers use these products [27-29]. In Karachi, Pakistan, 40% of the populations have used chewable betel, areca, and tobacco products in their daily life [30]. The overall prevalence of the use of the above products in men and women was 50.3 and 28.5%, respectively. A study conducted among school children in Karachi, Pakistan, reported that more than 74% of students used chewable products on a daily basis [31]. According to a report in 2006, the general use of *paan*, *chaalia*, *gutkha*, *naswar*, and *toombak* was determined to be 34.3, 34.7, 46.0, and 50.0% in the Sindh, Punjab, Pathan, and Mohajir provinces, respectively [19].

The prevalence of *paan* and *gutkha* has been reliably documented, although fluctuating results have been reported in investigations of specific communities. A study conducted one decade previously found that 46% of the residents of Karachi, Pakistan consumed *gutkha* habitually [32]. Similarly, in another study it was found that 35% of the patients visiting a health care center in Karachi, Pakistan, were habitual consumers of *paan*, *gutkha*, and/or other smokeless tobacco products [17].

### Tanzania

In Tanzania, 7% of the native inhabitants were found to use *gutkha* on a daily basis [41]. In addition to *paan* and *gutkha*, other risk factors were found to be involved in the development of oral cancer, as shown in Table 2 [21,42,43].

### Cambodia

In Cambodia, most users add tobacco to quid, and another practice is to rub it into the gum after chewing betel quid. The consumers of smokeless tobacco were mostly elder women [44]. In an epidemiological study, it was reported that 32.6% of women and 0.8% of men above 15 years of age chewed betel quid. Most of the men were approximately 50 years of age, and most of the women chewers were over the age of 39 in that study. Overall, smoking was prevalent in men (43%), but rare in women (4.5%) [45].

In the Pacific island of Palau, areca nut is chewed in a green unripe state, with other spices and flavoring ingredients. A population-based study conducted in 1991 revealed that 80% of women and 70% of men chewed areca nut or betel quid, 80% of whom included tobacco in the betel quid [46].

### Indonesia and Malaysia

The use of smokeless tobacco is different in these countries. In Indonesia, first the betel quid is chewed, and finally a fine-cut tobacco is used to clean the teeth, while keeping it in the mouth for a moment [8]. In Malaysia, use of *paan* and *gutkha* is high among some native groups, who use betel quid with tobacco.

### Usage in migrant communities

The high risk of oral cancer among migrants in the UK is due to the massive consumption of betel quid and other smokeless products. People who have migrated from Pakistan, India, Bangladesh, and Sri Lanka to the UK are the major communities in which betel quid use is widespread [47]. Various studies have found that over 80% of adults from Bangladesh in London used betel quid and other products, with no gender differences. The use of tobacco, in smoking or in smokeless forms, is common among men and women [48,49]. The usage of these products is becoming well known in these communities. Studies conducted on betel quid and tobacco use among South Asian emigrants to Western countries have pointed out the lack of awareness of oral cancer risk regardless of gender, age, national group, and social class. Individuals tend to be more aware of smoking, but not attentive to other habits that pose a risk of oral cancer, such as *paan* and *gutkha*.

## MECHANISM OF GENOTOXICITY AND CARCINOGENICITY

**Figure 2.**
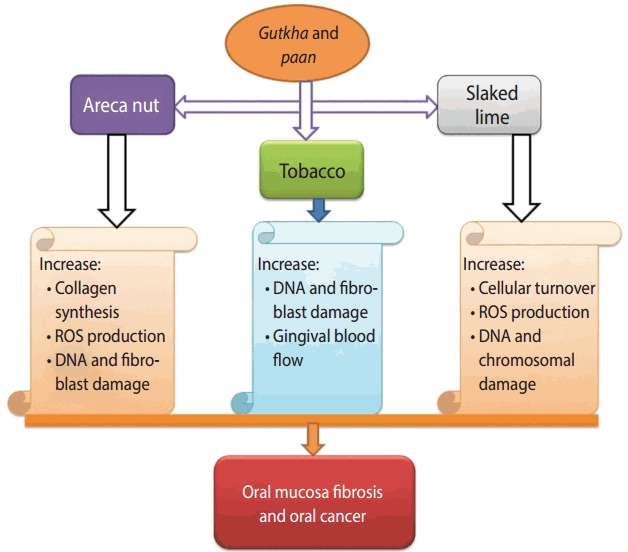
Roles of *paan* and *gutkha* in oral submucous fibrosis [42,43]. ROS, reactive oxygen species.

*paan*, *gutkha*, and *zarda* are taken by mouth, chewed, sucked, or applied to the teeth and gums. The World Health Organization has classified smokeless tobacco products as human carcinogenic compounds, in particular tobacco-specific nitrosamines, which account for 76 to 91% of the total N-nitroso compound (NOC) burden [14]. These products have been associated with oral and pancreatic cancers, cardiovascular disease, periodontal disease, asthma, and deformities in the women reproductive system [1]. The mechanisms of *paan* and *gutkha* that have been proposed for humans are summarized in Figure 2 [42,43]. Studies have shown that tobacco users who include slaked lime in betel quid or with areca nut experience carcinogenic and genotoxic effects in human oral epithelium cells. These products generate reactive oxygen species (ROS) in the oral cavity of chewers [50]. A study conducted in India reported that the greatest extent of DNA disruption was observed in *gutkha* consumers who smoked, with the following order of the extent of DNA denaturation: *gutkha*+smoking>*paan*+ smoking>*gutkha* only>*paan* only> smoking only> no tobacco use [51].

The different ingredients used in *paan* and *gutkha* have their own detrimental effects, such as catechu, which consists of tannin and polyphenols, which have a high tendency to cause esophageal cancer and are characterized by mutagenicity and clastogenicity [52-54]. The lime (calcium hydroxide) used can result in an alkaline pH, triggering ROS release and causing irritation of the oral mucosa and hyperplasia [55]. Areca nut consists of phenolic compounds, and tobacco releases various nitrosamines in the mouth that are responsible for proliferative abrasions and damage to DNA and fibroblasts [42,56].

**Figure 3.**
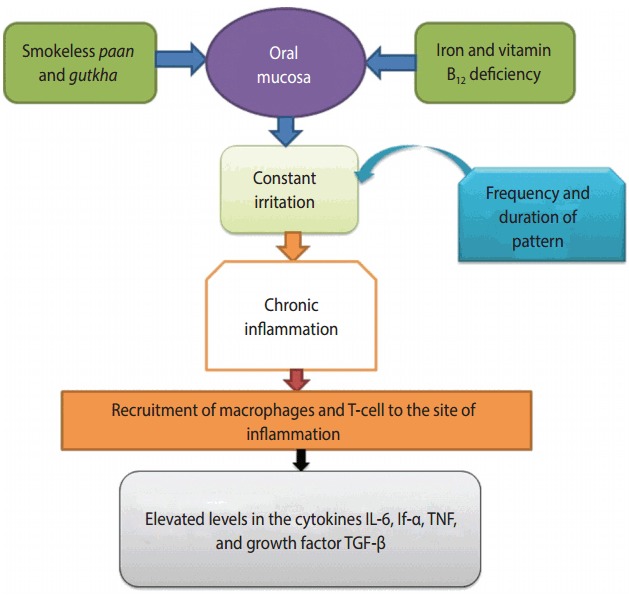
Initial events in the pathogenesis of mouth cancer [56,63]. IL-6, interleukin-6; If-α, interferon-alpha; TNF, tumor necrosis factor; TGF-β, transforming growth factor-beta.

A Mexican study conducted in 2006 indicated that single-cell gel electrophoresis is a safe method of determining DNA damage in human populations [57]. When the amount of ROS production in cells is increased in the presence of a normal detoxification system, oxidative stress leads to cellular damage, along with DNA damage [58]. DNA destruction may occur in the form of doublestrand or single-strand DNA breaks [59]. Areca nut is the main component of *paan* and *gutkha*, while the areca nut used in *gutkha* leads to OSMF [60]. The incidence of micronuclei (MN) was observed among OSMF patients who chewed *gutkha* [51]. Studies have shown that ROS production triggered OSMF [61]. NOCs extracted from areca nuts, which contain the active substance 3-(methylnitrosamino)proprionitrile, have been found to cause genotoxicity and cytotoxicity responsible for tumors in the buccal cavity of smokeless tobacco consumers [62]. The long-lasting and frequent presence of *paan* and *gutkha* in the mouth around the gums leads to inflammation of the oral mucosa, which causes the activation of T-cells and macrophages, and ultimately the release of prostaglandins (PGs). PG production occurs in keratinocytes of the buccal cavity due to areca nut extract, and this plays a significant role in oral tissue fibrosis and cancer. Cytokines such as interferon-α, tumor necrosis factor (TNF), interleukin-6, and growth factor-like transforming growth factor-beta have been found to be produced at the sites of irritation [63]. These chemical substances make important contributions to OSMF and premalignancy (Figure 3) [53,63].

**Figure 4.**
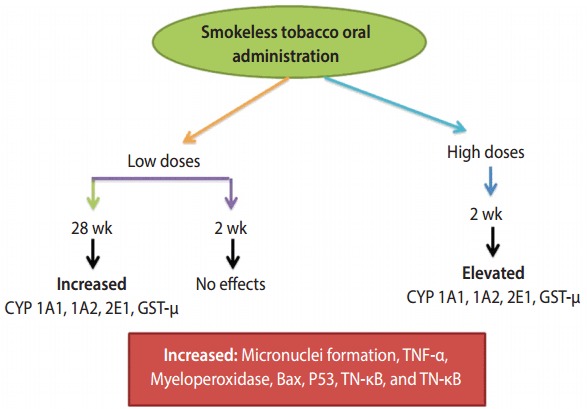
Molecular mechanism of *gutkha* [90,91]. CYP, cytochrome polymorphism; GST-μ, μ-glutathione-s-transferase; TNF, tumor necrosis factor.

Genotoxic effects occur from *paan* and *gutkha* mostly due to the presence of nitrosamine, as shown in Table 3. The nitrosamine in the chewers’ saliva undergoes nitration during betel quid chewing when it reacts with nitrite in the presence of a catalyst [42,64]. The nitrosamine in tobacco undergoes metabolic activation by cytochrome P450 enzymes, which may lead to the formation of N-nitrosonornicotine, a major carcinogen [65], which further leads to DNA damage and ultimately oral cancer. MN are small chromatin bodies that appear during cell division in the cytoplasm due to the condensation of whole chromosomes or acrocentric chromosomes; this is the only biomarker used to identify genotoxicity during sister chromatid exchange and chromosomal aberrations [66]. Multiple genes are involved in the breakdown of carcinogens, and the most frequently observed evidence has suggested that cytochrome polymorphisms (CYPs) affect the risk of oral cancer. Arecaidine, arecoline, and other similar ingredients of betel quid exist in minute quantities in human blood, and arecoline levels have been associated with the use of betel quid [67]. We have summarized the effects of CYPs, which are connected with the chewing of betel quid and lead to cancer of the oral cavity and pharynx, in Figure 4 [90,91]. Among the CYPs, CYP1A1 and CYP2E1 may trigger nitrosamines, which ultimately affect the mouth, potentially leading to malignant disorders [91]. In particular, μ-glutathione-stransferase was found to enhance TNF-α. Meanwhile, MN have been used for decades as a biomarker of genotoxic effects. The prolonged use of chewing or masticatory products such as *supari*, *paan*, and *gutkha* can lead to the development of different types of oral cancer. It is therefore necessary to evaluate the population at high risk due to using these products at high doses on a daily basis. *paan* and *gutkha* have been found to have carcinogenic effects in laboratory animals, causing tumors in various organs, such as the liver, mouth, pharynx, and larynx. *paan* acts as a cancer-promoting agent in mice [92]. A study was conducted in which mice that were fed with *paan* or *gutkha* exhibited tumors in the testes, ovary, liver, kidney, stomach, and lung. This suggests that *gutkha* and *paan* are not only carcinogenic for the oral cavity, but may also exert deleterious effects on other organs [93].

## DISCUSSION

Studies have shown that *paan* and *gutkha* comprise trace elements, such as magnesium, chlorine, calcium, sodium, manganese, copper, bromine, and vanadium. The copper content in these two products is more than other nuts consumed. The average content of copper in these processed products existing in betel nuts was 18± 9 µg/g, which is sufficient to exert deleterious effects on human health. According to the Indian Food Report, this concentration was 2.5 times greater than in raw betel nuts. The cellular metabolism of betel nuts and quid leads to the production of ROS, such as hydrogen peroxide and superoxide anion radicals, at a pH of more than 9.5 [43]. Saliva has the potential to inhibit the production of these ROS and other free radicals from the constituents of betel quid. Nonetheless, hydrogen peroxide and oxygen production occurs due to the alkaline pH that arises from the addition of slaked lime when chewing these products [73,94].

It seems that there is an association between oral inflammatory conditions and the age of individuals who use smokeless tobacco products [95]. However, *paan* and *gutkha* may also expose the consumers to other oral mucosal disorders at any stage of life. Javed et al. [32] reported that *gutkha* chewers 45-65 years of age had more periodontal inflammation. A study that investigated the effects of smokeless tobacco on blood flow response showed that tobacco significantly increased gingival blood flow, arterial blood pressure, and heart rate [96]. In addition, *paan* and *gutkha* chewers also have been found to exhibit reduced salivation and mucus formation, thereby reducing the normal microflora of the oral cavity and exposing the mouth to pathogens such as *Aspergillus* species [97]. For this reason, reduced salivation may also permit pathogens to accumulate in the supragingival and subgingival areas, thus increasing periodontal inflammation in *gutkha* chewers compared to non-chewers [98].

OSMF was an infrequent disease/condition during the 1960s and 1970s, with a prevalence in older individuals of approximately 0.1-0.5%. Subsequently, in the Indian subcontinent, the consumption of areca nut mixtures in products such as *paan* and *gutkha* has led to an epidemic of OSMF in adolescents [99,100]. Case-control studies in New Delhi, Maharashtra, Gujarat, Nagpur, and Bhavnagar have reported that more than 70% of cases of OSMF occurred in people under 35 years of age [2,69,99,100]. *paan* and *gutkha* chewers develop this condition more frequently than betel quid chewers. The incidence of OSMF is 75% after 4.5 and 9.5 years of use of *paan* and betel quid, respectively. The lack of betel quid leaf in *paan* and the higher levels of areca nuts may facilitate the development of OSMF in *paan* and *gutkha* users [21].

Areca nuts mixed with tobacco are not an etiological agent contributing to the development of submucous fibrosis. However, it is thought that the high occurrence of OSMF is due to its effect of enhancing addiction, leading to greater exposure to areca nut chewing. In a range of studies, OSMF was recognized as an extremely hazardous precancerous state. In a cohort study, 12,212 tobacco consumers with OSMF were at a higher risk of malignant transformation than tobacco users without any precancerous lesions [61,69,99]. Sufficient evidence has emerged that betel quid with tobacco, tobacco consumed by chewing with lime, betel quid with or without tobacco, and areca nuts are human carcinogens [69,73]. The use of these products has reached such a proportion that the government has no choice but to ban these products. In public health, banning such extensively used products is difficult and not ideal. In particular, betel quid should be banned, as *paan* and *gutkha* are items containing betel quid. The Central Committee of Food Safety of India has issued letters to the central government in support of a ban of betel quid, areca nut, *paan*, and *gutkha*.

The reasons for which the Central Committee of Food Safety consistently wants to ban the manufacturing and marketing of these products include: 1) adolescents and teenagers are becoming more addicted; 2) consumers develop OSMF, a precancerous condition, and cancer more quickly than smokers; and 3) women prefer smokeless tobacco due to the social disapproval of smoking and thereby become addicted to *gutkha*. Thus, in India, all legal evaluation processes necessary to ban *gutkha* nationally have been accomplished, but it continues to be manufactured and marketed in the black market and/or legally.

In the following states of India, *gutkha* and *paan* have been banned: Andhra Pradesh, Goa, Maharashtra (August 1, 2002), and Tamil Nadu (November 19, 2001). Certainly, *gutkha* producers are on the defensive [20]. *Gutkha* and *paan* producers have stated that these regulations were not catastrophic, as these products remain legal in the UK and Singapore, according to the *Times of India*. Thus, they continue to export *paan* and *gutkha* to the UK, Singapore [18,20], Middle East, Japan, and Australia, as well as across South Asia.

## CONCLUSION

In the present study, it was concluded that the extensive use of smokeless tobacco in different forms leads to OSMF, which potentially transforms into a malignant condition in all age groups. The genotoxic and carcinogenic effects of smokeless tobacco in the oral cavity are due to the production of ROS and free radicals. These free radicals and ROS damage the normal DNA and RNA, leading to genotoxicity and, eventually, oral cancer. Previously described mechanisms explain the induction, maintenance, and progression of OSMF due to *paan* and *gutkha*. Strict cessation of smokeless tobacco use and follow-up should be implemented to reduce the incidence of oral cancer. Widespread use of these and other products by children, as well as adolescents, is mostly due to their pleasant taste, low cost, and easy availability. Oral cancer rates are increasing due to use of these smokeless tobacco products, in particular among the lower socioeconomic levels that constitute the large majority of the population. It is therefore important to establish appropriate data management, monitoring, and evaluation systems. In addition, oral cancer control policies should be implemented to change the lifestyle and behavior of high-risk populations.

## Figures and Tables

**Table 1.** Some common forms of oral smokeless tobacco and their constituents

Common/native name	Ingredients	Countries/populations
*Toombak*	Sodium carbonate and tobacco	Sudan
*Shammah*	Tobacco, slaked lime, and ash	Saudi Arabia
*Naswar*	Tobacco, slaked lime, indigo, cardamom, oil, and menthol	Iran, Afghanistan, Pakistan, Central Asia
*Nass*	Tobacco, ash, cotton, and sesame oil	Iran, Afghanistan, Pakistan, Central Asia
*Mawa*	Areca nut, lime, and tobacco	India
*Gadakhu*	Tobacco and molasses	Central India
*Zarda*	Boiled tobacco	India and Arab countries
*Paan/betel quid*	Areca nut, betel leaf, slaked lime, spice, and catechu, with or without tobacco	Indian subcontinent, New Guinea, Southeast Asia, and South America
*Mishri*	Burned tobacco	India

**Table 2.** Summary of the risk factors for oral cancer in various Asian countries

Country	Associated risk factors	Study design	No. of subjects	Reference
Taiwan	Quid without tobacco, smoking, alcohol, heavy metals, HPV, SEC	Questionnaire-based	4906	[12]
Vietnam	Quid with tobacco	Visitors to cancer centers and institute of odontology, relevant publications in Vietnamese, interviews with betel quid vendors and individual betel quid users	_	[33]
Pakistan	*Paan, gutkha*, SEC, smokeless tobacco, bidi and cigarette smoking	Structured questionnaires	425	[19]
Sri Lanka	Betel quid, tobacco	Cross-sectional community-based study	1,029	[34]
Yemen	Cigarette smoking, smokeless tobacco, quid with tobacco	History-based study	649	[35]
India	Quid with or without tobacco, smokeless tobacco, alcohol, bidi and cigarette smoking, HPV, diet, SEC	Nested case-control design	1,692	[22]
Philippines	Quid, smoking	Case-control study	566	[36]
Thailand	Family history of cancer, alcohol, smoking, quid with tobacco	Case-control study	104	[37]
Malaysia	Malnutrition, HPV	Research analysis of frozen samples of oral tissue	210	[38]
Nepal	Bidi	Epidemiological study	Population aged more than 15 yr	[39]
Turkey	Malnutrition, alcohol, cigarette smoking, SEC	Case-control study	140	[40]

HPV, human papillomavirus; SEC, socioeconomic conditions.

**Table 3.** Studies of the genotoxicity of *paan* and *gutkha*

Study	Source	Endpoints
*Paan/gutkha* [67]	BC	MN
Tobacco [68]	EOMC	MN
Betel quid, areca, and tobacco [69]	BC and PBL	CA and MN
Tobacco [70]	EBC and PBL	MN and CA
*Paan* [71]	CC	CA, SCE, and MN
*Paan*/betel quid [72]	TRP	QBT
Lime [73]	EOMC	CT and MN
Slaked lime [74]	PMB	HE
Areca nut [75]	STS	Mutagenicity
Areca nut [76]	HBEC	CFFA, NRA, TRA
*Paan* [77]	Ovary cells	SCE and CA
Tobacco products [78]	ME, CTLE	Ames assay
Catechu [79]	Liver tissue	Ames assay
Arecoline [80]	BMC	SCE
*Paan* [81]	Mice	SCE
*Paan * [82]	PBL	SCE, CA, and MN
*Paan*, tobacco [83]	HOK	NHOKs
*Paan* [84]	BPL	MN, CA, and SCE
Tobacco [85]	VTF	DTT
*Paan* [86]	Mice	MA
Betel quid/*paan* [72]	TRP	QBT
Tobacco [87]	SNT	DTT
*Gutkha/paan* [88]	BMC	MN
Tobacco, areca nuts, and betel leaf [69]	PBL and BC	MN and CA
Tobacco [70]	PBL and EBC	CA and MN
*Paan* [89]	Rats	MEA

SCE, sister chromatid exchange; CA, chromosomal aberrations; STS, *Salmonella typhimurium* strains; PMB, popular band of paan; TRP, tobacco and its related products; CC, cultured cells; EBC, exfoliated buccal mucosal cells; PBL, peripheral blood lymphocytes; BC, buccal cells; MN, micronuclei; QBT, questionnaire about betel quid type; HBEC, human buccal epithelial cells; BMC, bone marrow cells; EOMC, exfoliated oral mucosal cells; ME, extract of masher; HOK, human oral keratinocytes; SNT, smoking and non-smoking forms of tobacco; CTLE, chewing tobacco and lime; MA, morphological abnormalities; NHOKs, normal human oral keratinocytes; CT, chemiluminescence technique; HE, histological examination; CFFA, colony-forming efficiency assay; VTF, various tobacco forms; NRA, neutral red uptake assay; TRA, trypan blue exclusion assay; MEA, marker of enzyme activities.
